# *GLI2* promoter hypermethylation in saliva of children with a respiratory allergy

**DOI:** 10.1186/s13148-018-0484-1

**Published:** 2018-04-11

**Authors:** Sabine A. S. Langie, Matthieu Moisse, Katarzyna Szarc vel Szic, Ellen Van Der Plas, Gudrun Koppen, Sofie De Prins, Tijs Louwies, Vera Nelen, Guy Van Camp, Diether Lambrechts, Greet Schoeters, Wim Vanden Berghe, Patrick De Boever

**Affiliations:** 1 VITO- Sustainable Health, Boeretang 200, 2400 Mol, Belgium; 2 Centre for Environmental Sciences, Hasselt University, Diepenbeek, Belgium; 3 Laboratory for Translational Genetics, Center for Cancer Biology, VIB and KU Leuven, Campus Gasthuisberg, Leuven, Belgium; 4 Proteinchemistry, Proteomics & Epigenetic Signaling (PPES), Department of Biomedical Sciences, University of Antwerp, Wilrijk, Belgium; 5 Environment and Health unit, Provincial Institute of Hygiene, Antwerp, Belgium; 6 Center for Medical Genetics, University of Antwerp and Antwerp University hospital, Antwerp, Belgium; 7 Department of Biomedical Sciences, University of Antwerp, Wilrijk, Belgium; 8 Department of Environmental Medicine, Institute of Public Health, University of Southern Denmark, Odense, Denmark

**Keywords:** DNA methylation, Saliva, Respiratory allergy, Illumina Methylation 450K BeadChip, GLI2

## Abstract

**Background:**

The prevalence of respiratory allergy in children is increasing. Epigenetic DNA methylation changes are plausible underlying molecular mechanisms.

**Results:**

Saliva samples collected in substudies of two longitudinal birth cohorts in Belgium (FLEHS1 & FLEHS2) were used to discover and confirm DNA methylation signatures that can differentiate individuals with respiratory allergy from healthy subjects. Genome-wide analysis with Illumina Methylation 450K BeadChips revealed 23 differentially methylated gene regions (DMRs) in saliva from 11y old allergic children (N=26) vs. controls (N=20) in FLEHS1. A subset of 7 DMRs was selected for confirmation by iPLEX MassArray analysis. First, iPLEX analysis was performed in the same 46 FLEHS1 samples for analytical confirmation of the findings obtained during the discovery phase. iPLEX results correlated significantly with the 450K array data (*P* <0.0001) and confirmed 4 out of the 7 DMRs. Aiming for additional biological confirmation, the 7 DMRs were analyzed using iPLEX in a substudy of an independent birth cohort (FLEHS2; N=19 cases vs. 20 controls, aged 5 years). One DMR in the *GLI2* promoter region showed a consistent statistically significant hypermethylation in individuals with respiratory allergy across the two birth cohorts and technologies. In addition to its involvement in TGF-β signaling and T-helper differentiation, *GLI2* has a regulating role in lung development.

**Conclusion:**

*GLI2* is considered an interesting candidate DNA methylation marker for respiratory allergy.

**Electronic supplementary material:**

The online version of this article (10.1186/s13148-018-0484-1) contains supplementary material, which is available to authorized users.

## Background

Respiratory allergies are increasing in frequency and severity and are the most common allergies in Europe and worldwide. The European Federation of Allergy and Airways Diseases Patients’ Associations (EFA) reported in 2011 that respiratory allergies affected around 20–30% of the European population [1]. More specifically, about 113 million European citizens suffered from allergic rhinitis and 68 million from allergic asthma in 2011. Zooming in on children as a vulnerable group, the International Study of Asthma and Allergies in Childhood (ISAAC) reported in 2009 that 10 to 20% of adolescents aged 13 and 14 in Europe were suffering from allergic rhinitis [2]. Genetic predisposition is an important risk factor for developing respiratory allergies, but the rise in prevalence happened within a too short time period to be explained by genetic changes. Epigenetic modulations, such as altered DNA methylation patterns in gene regulatory sequences, due to environmental exposures are a plausible mechanisms underlying the development and progression of respiratory allergies. Changes in the DNA methylation profile are expected to express themselves in altered gene and protein expression. Such changes contribute to the biological embedding of respiratory allergy during critical developmental phases. In this context, Bégin and Nadeau noted that DNA methylation changes in several loci are associated with the allergy phenotype and environmental exposures [3].

There is an increased interest in the discovery of DNA methylation markers that can differentiate individuals with a respiratory allergy from healthy subjects [4–20]. A number of studies focus on birth cohorts and describe the identification/confirmation of DNA methylation markers, either via an epigenome-wide or gene-targeted approach, in peripheral blood of children with respiratory disorders like wheezing or (allergic) asthma [10–17]. Blood-based DNA methylation analyses involved mostly the follow-up of school-age children, while we are aware of only three reports that describe DNA methylation markers in relation to allergic asthma and wheezing in peripheral blood from pre-school children around 4 years of age [14, 15, 17]. More recently, it has been shown that saliva can be used as biofluid to perform gene-targeted DNA methylation studies [19, 21] as well as genome-wide DNA methylation analysis [22–24]. In children, especially at pre-school age, analysis of saliva may be advantageous over a blood analysis, which is invasive and kept to a minimum for study compliance and ethical reasons. We demonstrated the feasibility of assessing DNA methylation patterns in saliva using genome-wide methylation analysis and observed that patterns were consistent with those in blood; the methylation status of about 96% of the cg-sites was comparable between peripheral blood mononuclear cells (PBMC) and saliva [25]. In addition, we identified differential methylated gene regions (DMRs) in a case-control study with adults having a respiratory allergy [25].

In the current study, we aimed at discovery and confirmation of DMRs in saliva of children with respiratory allergy when comparing them to healthy control subjects.

## Methods

### Study design and sample collection

The Flemish Environment and Health Studies (FLEHS) were established for following internal exposure of the general Flemish population in Belgium to environmental chemicals and the associated health effects [26, 27]. The FLEHS1 birth cohort enrolled 1196 mother–child pairs between September 2002 and February 2004 via 25 maternities across Flanders, covering 20% of Flanders’ area and 65 different municipalities. Details of the study and recruitment protocol have been previously reported [28]. During follow-up of the cohort at 10 years of age (*n* = 595), information on the allergy status of the children based on the ISAAC questionnaire [29] was collected. A sub-group (*n* = 99) of those followed-up at age 10 years agreed to donate saliva and blood samples and filled out an additional questionnaire at the age of 11 years. Unstimulated collection of saliva samples (2 mL) was performed using Oragene DNA OG-500 self-collection kits (DNA Genotek, Ottawa, Canada). The saliva samples were kept at room temperature until DNA extraction. Blood samples (10 mL) were collected in EDTA tubes (BD Vacutainer®, BD, Plymouth, UK) and kept less than 2 h at room temperature until further processing. Plasma was collected after centrifugation at 800×*g* for 5 min and stored at − 20 °C for further clinical characterization.

In the second campaign, FLEHS2, 255 newborn-mother couples were recruited across the five provinces of Flanders between August 2008 and July 2009 via 10 randomly selected maternities. The study protocol has been described elsewhere [30]. The participation criteria where similar to those for FLEHS1, with the exception that mothers should have resided in Flanders for 10 years in order to be included. Health data and lifestyle information were obtained via questionnaires, including the allergy status based on the ISAAC survey. In a follow-up at 5 years of age, 78 children provided saliva samples and completed a short ISAAC-based survey mainly focused on allergy symptoms. At the age of 7 years, the participants completed a more extensive questionnaire capturing health and lifestyle data, with the main aim to confirm their allergy phenotype.

### Determining IgE sensitization status and defining cases

For FLEHS1, plasma samples of the 11-year-old children were analyzed using an ImmunoCAP Phadiatop test (Thermo Fisher; performed by the medical lab AML, Antwerp) to determine specific IgE sensitization status for a mix of airborne allergens: birch, cat, dog, house dust mites (*Dermatophagoides pteronyssinus*), and grass pollen. A cutoff value 0.35 kUA/L was used to define an IgE-positive status [31]. Note that children occasionally express respiratory allergy symptoms due to food allergies. To rule these out, blood samples were analyzed for a food allergen mix (ImmunoCAP Fx5mix; egg, cow milk, fish, wheat, peanut, and soy allergens; available from Thermo Fisher and performed by the medical lab AML, Antwerp).

Twenty-six children participating in the current sub-study of the FLEHS1 birth cohort were considered to have a respiratory allergy if they reported (either self-reported or doctor’s diagnosed) at least one respiratory allergy symptom (occurrence of asthma, hay fever, other types of rhinitis, wheezing, or runny nose, in the past year and ever; as questioned in accordance with the ISAAC questionnaire [29]) and Phadiatop IgE ≥ 0.35 kU/L. Twenty control subjects were assigned that did not report any (doctor’s diagnosed) allergy symptoms, and Phadiatop and FX5 IgE < 0.35 kU/L.

Due to the unavailability of blood samples to measure IgE levels in the FLEHS2 cohort, cases and controls were identified as (1) cases = doctor’s diagnosed and/or self-reported respiratory allergy symptoms (*n* = 19) and (2) controls = no self-reported and/or diagnosed allergies plus ≤ 1 reported incidence of family history for allergy (*n* = 20). Both the questionnaires completed at age 5 and age 7 were consulted to define cases and were checked for misreporting.

Since the Flemish birth cohorts were designed as environmental health surveys, and less as a clinical study into allergy, all respiratory allergy subtypes (e.g., allergic asthma, rhinitis, hay fever, house dust mite) were combined into one group of respiratory allergy cases to increase the sample size and power of the study.

### DNA extraction and bisulfite treatment

Genomic DNA was extracted from saliva using the Oragene PrepIT kit (DNA Genotek, Ottawa, Canada) according to the manufacturer’s instructions. About 500 ng of gDNA was bisulfite converted using the EZ DNA methylation kit (Zymo Research, Cambridge Bioscience, Cambridge, UK) according to the manufacturer’s instructions. Bisulfite conversion and quality control was performed as previously described [25].

### Infinium HumanMethylation450 BeadChip Array and data processing

Genome-wide DNA methylation profiles were generated with Infinium HumanMethylation450 BeadChip Array (Illumina, San Diego, CA, USA) according to the standard Infinium HD Assay Methylation Protocol Guide (Part #15019519, Illumina). The BeadChip images were captured using the Illumina iScan. The raw array data were uploaded to the Gene Expression Omnibus (GEO) database and have accession number GSE110128.

Raw data analysis, QC, normalization, cell-count estimation, and methylation *β* value conversions were performed using the R-packages minfi [32] and IMA [33]. In brief, the raw Red/Green channel data from the 450K-lllumina methylation array were read by the “read.450k.exp” function, converted to methylation values by “preprocessRaw” and subsequently normalized using “preprocessSWAN,” an implementation of the Subset-quantile Within Array Normalization (SWAN) normalization procedure [34]. Principal component analysis and unsupervised clustering were used to check for sample outliers. All samples passed quality controls and were loaded into the R-package IMA for further processing/filtering. Samples having > 75% of CpG sites with a detection *p* value > 1e−05 were removed (all samples passed this filter). Cg-probes with a detection *p* value greater than 0.01 in all samples and cg-probes on the X and Y chromosomes were removed.

The normalized *β* values of the 450K BeadChip data were converted to *M* values (*M* = log2(*β*/(1 − *β*))) for statistical analysis [35] and differential methylation between samples (respiratory allergy cases vs. healthy controls) was estimated with linear models using R-package Limma [36]. Gender, batch effect, and differences in cell composition were included as covariates. Resulting *p* values were corrected for multiple testing using the Benjamini-Hochberg procedure (*p*_adj_). Results in tables and figures are presented as median *β* values ± standard deviation.

To estimate the proportion of various salivary cell types, the statistical deconvolution method described by Houseman et al. and implemented in “minfi” as the “estimateCellCountsMset” function was used [37]. Reference methylomes from leukocyte subtypes were obtained from the study of Reinius et al. [38]. Buccal epithelial cell reference methylomes were obtained from the GEO dataset GSE48472 [39].

### iPLEX MassArray analysis

Differentially methylated CpG sites in the identified DMRs were further confirmed using iPLEX MassArray analysis by Agena Bioscience (Hamburg, Germany) according to the manufacturer’s protocol (iPLEX® Pro Application Guide, SQNM-USG-CUS-030 Rev 3.0; CO-12-274, 2012). Briefly, the iPLEX technique targets individual CpG sites and allows simultaneous analysis of multiple CpGs in a single-well reaction. PCR- and extension primers (probes) were automatically developed with SEQUENOM’s MassARRAY Designer Software for each CpG that had to be investigated, and were run as 7 multiplexes and 1 singleplex. One microliter of the bisulfite-treated DNA (5–10 ng/μL) was added to 100 nM of a primer mix (500 nM each primer) in a 5-μL reaction. Amplification was carried out as follows: 95 °C for 2 min, then 45 cycles of 95 °C for 30 s, 56 °C for 30 s, and 72 °C for 60 s, followed by 72 °C for 5 min. After PCR, the unincorporated dNTPs are neutralized with SAP treatment. Resulting primer extension products were Resin treated to remove access salt and were analyzed with MALDI-TOF MS as each primer and its extension products have a unique molecular mass. The peak heights are indicative for the methylation status. The spectra were acquired using the MassARRAY® Analyzer instrument, and raw data were processed with TyperAnalyzer software according to the MassARRAY® Analyzer User’s Guide.

### Statistical analysis

DMRs were identified with comb-p analysis [40], using the list of uncorrected *p* values for all CpG sites as calculated from the differential methylation analysis together with their chromosomal location. This statistical procedure combines adjacent *p* values, performs false discovery adjustment, and finds regions of enrichment. For these regions, it assigns a combined Stouffer-Liptak *p* value based on the uncorrected *p* values and finally corrects for multiple testing using the Sidak correction, yielding adjusted *p* values (*p*_adj_). The generated output is a list of regions that are differently methylated, and an aggregated, adjusted *p* value is assigned to each region [40]. A region with an adjusted *p* value < 0.05 was deemed differently methylated.

**Fig. 1:**
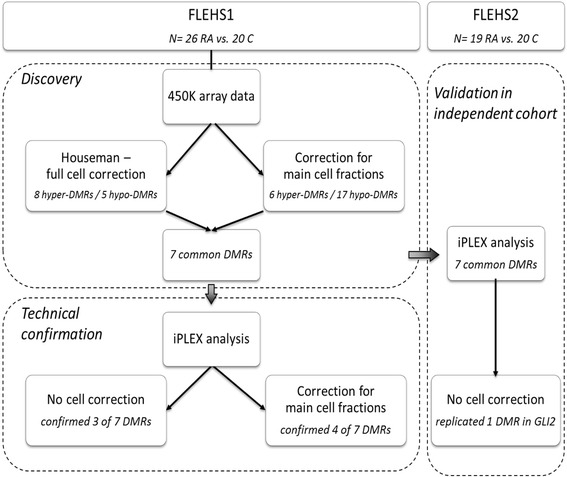
Schematic overview of the various cell correction methods applied. The Houseman cell correction involved correction for the various leukocyte subtypes and buccal cell methylomes (GEO GSE35069 and GSE48472, respectively), while the correction for the main cell fractions only involved correcting for the granulocyte and buccal cell fractions

Two different methods were applied to correct for differences in cell proportions in the FLEHS1 study. In a first step, we applied the full reference-based deconvolution method, according to Houseman et al. (as described above), and corrected our methylation data for the following cell proportions: granulocytes, CD4+ T cells, CD8+ T cells, B cells, monocytes, NK cells, and buccal cells. In parallel, normalized *β* values were corrected for the buccal and granulocyte cell fractions only (using GEO GSE35069 and GSE48472 as granulocyte and buccal reference methylomes). The estimated granulocyte and buccal cell fractions were also used to correct the FLEHS1 iPLEX data. For the FLEHS2 study, no Illumina data were available and cell counts could not be estimated. As such, for comparison of the iPLEX data between FLEHS1 and FLEHS2, non-corrected data were analyzed with SPSS statistics. Figure 1 gives an overview of the performed data analysis and various cell correction methods.

Using SPSS statistics, correlations between methylation in saliva samples detected by 450K BeadChip or iPLEX MassArray were analyzed via the Spearman correlation coefficient (*ρ*). The iPLEX MassArray data were analyzed by General Linear Models, Univariate Analysis of Variance to identify statistical differences in methylation levels of the DMR between respiratory allergy cases and healthy controls. The methylation level of a DMR was calculated as the mean methylation of the CpGs in that region. The models for the analyses on the FLEHS1 data were corrected for buccal and granulocyte cell count, gender, and batch in which samples were analyzed (since there was a gap of a year between the analysis of the 2 batches). The FLEHS2 samples were analyzed as one batch, and the models using these data were only corrected for gender. A *p* value below 0.05 was considered statistically significant. A DMR region was considered “confirmed” when the region reached statistical significance and a delta beta difference in the same direction as the original finding.

### Functional analysis

Biological interpretation and identification of interactions between molecules using network analysis via Ingenuity Pathway Analysis (IPA) was performed (http://www.ingenuity.com/). The freely available “ChromHMM from ENCODE/Broad” tool [41–43], as part of the UCSC hg19 browser, was used to visualize the chromatin state segmentation of the *GLI2* DMR. ChromeHMM uses a multivariate Hidden Markov Model (HMM) and integrates multiple chromatin datasets such as ChIP-Seq data of various histone modifications to discover de novo the major re-occurring combinatorial and spatial patterns of marks. Estimation of gene expression values was computed based on the number of reads which map per kb of exon model per million mapped reads (RPKM) for each gene, for each tissue or sample, as generated by the Genotype-Tissue Expression (GTEx) project (derived from Ensembl genes hg18, UCSC genome browser) [44, 45].

## Results

### Characteristics of the study population

In the FLEHS1 sub-cohort, the children with respiratory allergy (17 boys and 9 girls) mainly reported rhinitis (defined as runny nose without suffering from a cold; *N* = 23), among which 14 reported also to suffer from hay fever and 8 had asthma (*N* = 8; 4 in combination with hay fever). Two children reported only hay fever without the signs of asthma or rhinitis/runny nose (so total *N* = 16 for hay fever). The median (±SE) Phadiatop IgE level detected for the allergy cases was 29.5 ± 7.0 kU/L and a mean FX5 IgE level of 0.14 ± 3.9 kU/L. Preventive medication (i.e., inhalation of (cortico)steroids, sometimes in combination or oral ingestion of a leukotriene receptor antagonist or antihistamines) was taken daily by 5 of the cases, while 11 children only used medication (β2 adrenergic receptor agonist and/or corticosteroid inhaler/nasal spray, sometimes in combination with oral intake of antihistamines, or homeopathic treatment) when experiencing an upsurge. The healthy control group consisted of 8 boys and 12 girls; median (±SE) Phadiatop and Fx5 IgE levels were 0.00 ± 0.04 and 0.00 ± 0.02, respectively.

Among the 5-year-old children with a respiratory allergy in the FLEHS2 sub-cohort, there were 8 boys and 11 girls, and for 12 of the children, it was reported that one or both of their parent suffered from 1 or more respiratory allergies. By the age of 7, there were 2 cases of asthma (1 in combination with hay fever), 12 reported symptoms of rhinitis, and 4 had a wheezing phenotype. In addition, 5 children suffered from house dust mite allergy, including 3 out of the 12 children who reported rhinitis/runny nose and the asthma case who also reported hay fever. Preventive medication (oral intake of antihistamines) was taken daily by 2 of the cases, while 2 other only used medication (oral intake of antihistamines combined with a steroid inhaler or nasal spray) when having an upsurge. The control group consisted of 11 boys and 9 girls, among which 9 children reported to have one case of respiratory allergy in their family.

### Discovery of DMRs in FLEHS1 birth cohort

**Fig. 2:**
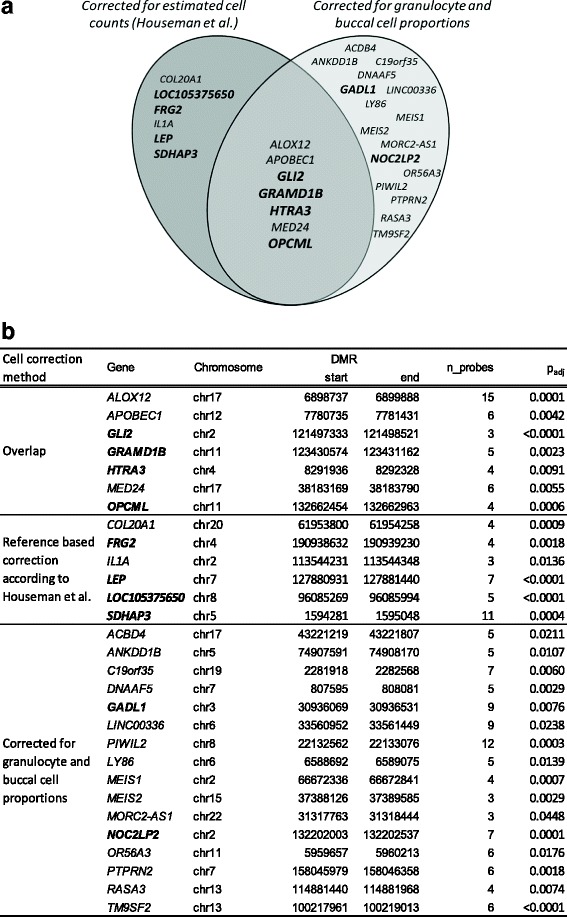
Overview of identified DMRs in FLEHS1 birth cohort. **a** Seven gene regions showed an overlap between the DMRs identified via cell correction according to Houseman et al. and those after correction for the granulocyte and buccal cell proportions. Hyper-methylated gene regions are shown in bold; all other DMRs were hypo-methylated in respiratory allergy cases compared to controls. **b** The location of the DMRs in the genome and the number of significantly different methylation probes were analyzed (corresponding to CpG-sites) in these gene regions

Following quality filtering and normalization of the Illumina 450K data, 470,562 cg-probes (96.9%) from the original 485,512 probes were kept for downstream data analyses. Comb-p analysis revealed 13 DMRs (Fig. 2) between respiratory allergy cases and controls when correcting for differences in cell composition. The parallel analysis, involving correction of the data only for the main salivary cell fractions (i.e., buccal cells and granulocytes), revealed 23 DMRs in respiratory allergy cases compared to control subjects. The 7 DMRs in common between the 2 data analysis strategies were selected for further confirmation. Four of these DMRs were hypermethylated, and 3 DMRs were hypomethylated in children with a respiratory allergy compared controls (Fig. 2, Table 1).

**Table 1:** iPLEX MassArray confirmation of the 7 DMRs identified with 450K array in FLEHS1 saliva samples

Gene	450K array data				iPLEX MassArray data				
	Allergy	Control	Delta beta	*p*_adj_^a^	Allergy	Control	Delta beta	*p* ^b^	*p* ^c^
	Beta ± SD	Beta ± SD			Beta ± SD	Beta ± SD			
*ALOX12*	0.35 ± 0.10	0.39 ± 0.07	− 0.044	0.0001	0.24 ± 0.08	0.26 ± 0.05	− 0.021	0.109	0.102
*APOBEC1*	0.84 ± 0.09	0.86 ± 0.07	− 0.022	0.0042	0.76 ± 0.23	0.83 ± 0.20	− 0.068	0.109	0.102
***GLI2***	0.74 ± 0.10	0.73 ± 0.07	0.009	< 0.0001	0.63 ± 0.12	0.61 ± 0.09	0.020	0.034*	0.035*
***GRAMD1B***	0.16 ± 0.04	0.15 ± 0.03	0.010	0.0023	0.10 ± 0.03	0.08 ± 0.03	0.016	0.002*	0.002*
***HTRA3***	0.66 ± 0.07	0.65 ± 0.05	0.013	0.0091	0.56 ± 0.07	0.53 ± 0.06	0.030	0.036*	0.011*
***MED24***	0.53 ± 0.02	0.54 ± 0.02	− 0.011	0.0055	0.40 ± 0.04	0.40 ± 0.03	− 0.001	0.328	0.036*
*OPCML*	0.66 ± 0.07	0.65 ± 0.09	0.003	0.0006	0.50 ± 0.08	0.48 ± 0.12	0.021	0.125	0.140

^a^Corrected for estimated cell counts (according to Houseman et al.), batch, and gender and adjusted for multiple testing

^b^Corrected for batch and gender

^c^Corrected for estimated buccal and granulocyte cell fractions, batch, and gender

Genes given in bold are significantly differentially methylated (*p <0.05)

### Confirmation of 450K methylation data by iPLEX MassArray

Analysis using iPLEX MassArray showed significant positive correlations with the Illumina 450K array data for the 7 DMRs (*p* < 0.0001; *ρ* = 0.89–0.95, except for *APOBEC1* (*ρ* = 0.55), *GRAMD1B* (*ρ* = 0.80), and *MED24* (*ρ* = 0.64)). The same polarity of the direction of the methylation changes was observed for all the studied DMRs (Table 1). However, when comparing respiratory allergy cases with control subjects, we were only able to confirm 3 of the 7 DMRs, those located in the genes *GLI2*, *GRAMD1B*, and *HTRA3*. In parallel, a cell correction analysis was performed, correcting the iPLEX data for the granulocyte and buccal cell proportions as identified from the 450K array data. These data analysis resulted in a confirmation of 4 of the 7 DMRs, located in *GLI2*, *GRAMD1B*, *HTRA3*, and *MED24* (Table 1).

### Verification of DMRs in the independent birth cohort FLEHS2

The DMRs that were identified in FLEHS1 samples were further investigated using iPLEX MassArray in saliva samples obtained from children taking part in the FLEHS2 cohort. Two significant DMRs in the *GLI2* and *GRAMD1B* genes from respiratory allergy cases versus control subjects were confirmed in FLEHS 2 (Table 2). The DMR in *GLI2* was confirmed with a 7% hypermethylation in children with a respiratory allergy compared to control subjects, whereas *GRAMD1B* showed the opposite methylation change (2% hypomethylation in cases compared to controls) when compared to the results obtained for the FLEHS1 cohort (1.6% hypermethylation in cases compared to controls).

**Table 2:** iPLEX MassArray verification of the seven identified DMRs in the independent birth cohort FLEHS2

Gene	FLEHS2 iPLEX MassArray data			
	Allergy	Control	Delta beta	*p* ^a^
	Beta ± SD	Beta ± SD		
*ALOX12*	0.24 ± 0.05	0.26 ± 0.06	− 0.016	0.443
*APOBEC1*	0.79 ± 0.17	0.82 ± 0.16	− 0.036	0.348
***GLI2***	0.72 ± 0.08	0.65 ± 0.10	0.071	0.017*
***GRAMD1B***	0.08 ± 0.03	0.10 ± 0.03	− 0.020	0.035*
*HTRA3*	0.59 ± 0.08	0.55 ± 0.06	0.041	0.057
*MED24*	0.37 ± 0.04	0.39 ± 0.04	− 0.016	0.207
*OPCML*	0.47 ± 0.08	0.50 ± 0.10	− 0.030	0.317

^a^Corrected for gender

Genes given in bold are significantly differentially methylated (*p <0.05)

### Transcriptional activity and biological interpretation of the *GLI2* DMR

The hypermethylated DMR identified by the comb-p software in the *GLI2* gene is a combination of 3 hypermethylated cg-sites: cg00637745, cg13872898, and cg17870997. This DMR is located in an intragenic CpG shore, 707 bp upstream from CpG-island 56. According to the UCSC genome browser h19 (based on data from the Bernstein Lab at the Broad Institute—part of the ENCODE consortium), the region is enriched for H3K4Me1 and H3K27Ac histone marks (Additional file 1: Figure S1), which are associated with enhancers or enhanced transcription, respectively [46]. Indeed, based on the Chromatin State Segmentation database from ENCODE/Broad Institute ([42, 43]; available through the UCSC genome browser h19), several enhancers and promoters are located within the DMR in *GLI2*. More specifically, using available data from H1-hESC embryonic stem cells, cg00637745 and cg13872898 seem to be located at the start of or within a weak promoter, while cg17870997 is located at the beginning of an active promoter. When considering data from the normal human lung fibroblast cell lines (NHLF), cg00637745 and cg13872898 were indicated to be located in a weakly transcribed region and cg17870997 in a weak enhancer region. Data on the GM12878 lymphoblastoid EBV-immortalized cell line suggest that the DMR is a polycomb-repressed region. RNA-Seq data generated by the GTEx project revealed *GLI2* gene expression to be significantly expressed in lung (1.394 RPKM) and salivary gland (0.902 RPKM) tissues and repressed in blood cells (Additional file 2: Figure S2).

**Fig. 3:**
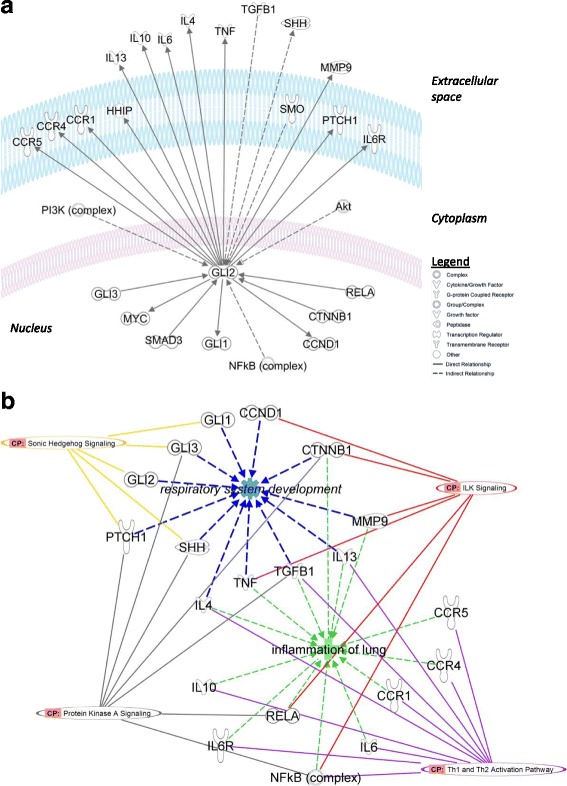
Network showing the interactions of GLI2 with other molecules and the associated functions. **a** The upper panel depicts a network of GLI2 with its most relevant up- and downstream molecules, while the lower panel **b** gives an overview of the associated pathways, functions, or diseases

The IPA tool was used to grow a network for GLI2, visualizing interactions with up- or downstream molecules. The network was simplified by keeping only the nearest neighbors of GLI2 (Fig. 3a) and overlaying the network with biological functions (Fig. 3b). According to the IPA Knowledge Base, the network included sonic hedgehog (SHH) signaling molecules (incl. GLI proteins) and key inflammatory molecules such as IL4, IL6, IL10, and IL13, as well as IL6 receptor and several chemokine receptors (CCR). Six molecules were linked to integrin-linked kinase (ILK) signaling, 10 molecules play a role in the Th1/Th2 activation pathway, and several transcription regulators (NFκB, RELA, GLI3) are involved in protein kinase A signaling. Interestingly, all molecules could be linked to 2 main functions: respiratory system development (incl. GLI family proteins, SHH, TGFB1, and cyclins) or lung inflammation (incl. cytokines and chemokine receptors).

## Discussion

This study identified hypermethylation in a CpG shore of the *GLI2* gene promoter, which discriminates school-age children with a respiratory allergy from healthy control children using saliva samples. The observations were made in a discovery case-control study (FLEHS1) and confirmed in an independent case-control study (FLEHS2). The discovery was initially performed with Illumina 450K arrays and confirmed with gene-specific iPLEX MassArray technology.

### *GLI2* DNA methylation differences in respiratory allergy

GLI2, together with GLI1 and GLI3, belongs to the GLI family of zinc-finger DNA-binding proteins [47, 48]. More specifically, it belongs to the type 2 zinc-finger protein subclass of the GLI family and is therefore characterized as transcription factor which bind DNA through zinc finger motifs. GLI2 is a key mediator of the SHH signaling pathway in vertebrates ([47–49]; Fig. 3). The SHH signaling pathway has an essential role in embryonic development and is critical for maintenance of tissue polarity [48]. Indeed, aberrant DNA methylation in the *GLI2* gene and other HH pathway members has been studied in relation to developmental disorders (including pre-eclampsia and Down syndrome) [50–54] and cancer [55–57]. Several GWAS studies indicate a role of HH pathway molecules in the etiology of asthma [58, 59], and the recent study by Murk and DeWan [60] reported a possible interaction between *GLI2* and the major asthma susceptibility gene *ADAM33*. However, as far as we know, there are no reports in the literature on differential methylation in *GLI2* in relation to respiratory allergy.

In the current study, we identified a DMR in the *GLI2* gene that distinguishes children with a respiratory allergy from healthy controls. According to UCSC genome browser and ENCODE data, this DMR is located in a CpG shore encompassing several enhancers and promoters (Additional file 1: Figure S1), suggesting that this region is involved in the active transcription of *GLI2*. Irizarry et al. previously observed strong inverse relationships between differentially methylated CpG shores and transcription of associated genes (incl. hypermethylation leading to reduced expression of the studied tissue-specific differentially methylated gene regions), supporting a functional role for CpG shores [61, 62]. In their study, this association was especially confirmed for shores located within 2 kb of an annotated transcriptional start site, but the possibility of additional regulatory functions for shores located in intragenic regions (as is the case for our identified *GLI2* DMR) was left open. In addition, according to the recently revised *GLI2* exon/intron structure nomenclature by Sadam et al. [63], our *GLI2* DMR under investigation appears to be located at an alternative transcription start sites (TSS) in exon III. These findings underscore the importance of the studied gene region in the regulation of *GLI2*.

Aiding in the discovery of the *GLI2* DMR in the current study was the use of alternative cell correction methods to reduce false positive hits and to account for possible differences in cell composition between the reference data generated using adults opposed to the children’s profiles studied in the current study. Correcting peripheral blood DNA methylation array data for differences in cell composition is generally performed according to the reference-based deconvolution method described by Houseman et al. [37]. For saliva, the most practical approach was to use blood plus buccal reference datasets [22, 25] to perform cell correction. Note that the Houseman correction does not discriminate between the various granulocyte subtypes, which might be important in future allergy studies because the proportion of eosinophils can change in individuals with allergy.

We previously identified with a reference-free deconvolution method [64] two underlying cell types in saliva that correlated significantly with the buccal and granulocyte cell fractions that were estimated to be the main cell type constituents in saliva according to the reference-based method [65]. A complementary approach was therefore to correct methylation array data for the buccal and granulocyte cell fractions only, in parallel to the full referenced-based deconvolution method. This enabled us to look at DMRs in common between both approaches and discovering true positive and biologically significant DNA methylation markers. The information on the cell fractions was also used to adjust the gene-targeted iPLEX methylation data. This is considered as strength of our study because it is not a common practice to correct gene-targeted methylation data for differences in cell proportions, mainly because data on cell counts (e.g., assed via flow cytometry) are usually not available or cannot be estimated in gene-targeted methylation studies.

A limitation of our study is that all respiratory allergy subtypes (e.g., allergic asthma, rhinitis, hay fever) were combined into one group of respiratory allergy cases. The reason is being that the Flemish birth cohorts were mainly designed as an environmental health survey. These studies were developed in context of general health monitoring and were not geared towards molecular studies into respiratory allergy. Hence, the number of cases with full clinical characterization is limited. Nevertheless, we used information from the ISAAC questionnaire and Phadiatop IgE levels to divide the cases further into specific respiratory allergy subtypes. Although the numbers per group were quite low to perform robust statistical analysis, we observed that hypermethylation of the *GLI2* DMR was mainly associated with rhinitis in the FLEHS1 cohort (Additional file 3: Table S1) and with house dust mite allergy in the FLEHS2 cohort (Additional file 3: Table S2). However, considering the small sample sizes in our study, the complex spectrum of respiratory allergy phenotypes, and other factors possibly modulating DNA methylation (e.g., medication use), we warn for extrapolation of our findings.

In general, gene hypermethylation is assumed to lead to decreased gene and protein expression. However, concerning the complexity of HH signaling with its feedback loops and the bi-functional nature of GLI2, it is hard to give a straightforward interpretation of the biological/phenotypic consequences of the *GLI2* DMR hypermethylation. GLI proteins bind DNA at consensus GLI-family binding sites and directly modulate to the expression of HH target genes, some of which are involved in HH pathway feedback (e.g., *GLI1*, *PTCH1*) [48]. In addition, as determined by posttranscriptional and posttranslational processing, GLI2 as well as GLI3 contains an amino-terminal repressor (R) and a carboxyterminal activator (A) domain. Thus, the ratio of GLIA relative to GLIR in the cell regulates the GLI-dependent transcription of target genes. GLI1 lacks the repressor domain and acts thus as an activator. GLI2A can act as a repressor and activator of transcription, whereas GLI3 has primarily a repressor function [66]. According to Sadam et al. [63], our *GLI2* DMR is located near a TSS and, considering the various possible splice variants, the identified DMR should be involved in the transcription of both GLI2A and GLI2R. So regardless the complexity of GLI2-dependent transcription, in the sections below, we describe the plausible pathways via which *GLI2* hypermethylation could increase the risk of respiratory allergies.

### The role of GLI2 in T-helper cell differentiation

HH signaling mediated by the HH-responsive transcription factor GLI2 has multiple roles in T-lymphocyte development and differentiation. In recent research, Furmanski et al. [47] demonstrated in Gli2A mice (carrying a truncated form of Gli2 that acts as a permanent transcriptional activator of Hh target genes) that (1) GLI2A promotes Th2 differentiation (via IL4 and GATA3) in T cells, (2) *Il4* is a novel target gene of Gli2-dependent Hh signaling, and (3) an allergic disease pathology was observed in house dust mite-treated Gli2A mice—showing increased eosinophil recruitment indicating enhanced inflammation, in parallel to a higher proportion of CD4^+^ T cells in the lung producing IL4 and IL-13, and goblet cell hyperplasia. In addition, the same group recently showed that GLI activity is induced in multiple leukocytes in a murine model of asthma and that expression of GLI2R decreases the recruitment of CD4+ Th2 cells to the allergic lung [67]. However, they reported that the allergic disease pathology was not significantly different from the WT. Still, both GLI2A and GLI2R might be closely involved in immune system development and Th2-mediated diseases. In addition, Kugler et al. indicated that it is ultimately the balance between GLI2A and GLI3R accumulation and the resulting target gene transcription in the nucleus that influences the pathway output [68].

Alternatively, GLI-dependent transcription can also be triggered by non-HH ligands, such as transforming growth factor β (TGF-β) [69]. Interestingly, associations between GLI2 and TGF-β1 were observed in our IPA network analysis (Fig. 3). In addition, knockdown of *GLI2* in human CD4+ CD25 regulatory T cells showed that GLI2 is also necessary for TGF-β1 transcription. In its activated state, GLI2 can bind on six assumed GLI binding sites in human TGF-β promotors. Thus, GLI2 can enhance TGF-β1 transcription in naïve CD4+ T cells [70]. TGF-β is produced by several immune-related cell types including CD4+ CD25 regulatory T cells, macrophages, and fibroblasts [70, 71]. TGF-β has been reported to play a key role in asthma because it mediates leukocyte chemotaxis to the lungs and thus genesis and maintenance of an inflammatory response. Furthermore, it was suggested that high TGF-β levels in the airways correlate with asthma severity, with eosinophils representing about 70–80% of all cells expressing TGF-훽1 in these patients’ airways (reviewed by [71]). In an alternative hypothesis, *GLI2* hypermethylation could lead to reduced expression of TGF-β1, resulting in enhanced immune Th-cell differentiation and pro-inflammatory cytokine production. However, TGF-β1 has also been reported to have anti-inflammatory and immunosuppressive properties, as reflected by the inhibition of immune cell differentiation (Th1 and Th2 cells and B cells) and cytokine production (IFN-γ and IL-2) [71]. Moreover, TGF-β has been described as a key molecule in the repair of the airway epithelium in allergic diseases such as asthma and allergic rhinitis [71]. In addition, enhanced expression of GLI2 also modulates TCR repertoire selection and results in lower CD4+/CD8+ ratio [49]. Based on the anti-inflammatory role of TGF-β1, another hypothesis might be that *GLI2* hypermethylation could lead to reduced expression of TGF-β1, resulting in enhanced immune Th-cell differentiation and pro-inflammatory cytokine production.

Extensive research has been done to elucidate the role of regulators of the Th differentiation (transcription factors, cytokines, and other immune derived cell molecules), but little is known about the contribution of non-immune factors, such as HH pathway-associated genes like *GLI2*. A recent report demonstrates that hedgehog pathways and TGF-β signaling both converge to GLI2 [72]. The crosstalk of these signaling pathways and their role in respiratory allergic diseases requires further investigation.

### The role of GLI2 in lung development

GLI2 is natively expressed in the lung mesenchyme (mucus connective tissue) near the epithelial border, and it is important for proper epithelial mesenchymal signaling during early long development [73]. Estimation of gene expression (derived from Ensembl genes hg18, UCSC genome browser) showed reasonable expression of *GLI2* in the lung and salivary gland (Additional file 2: Figure S2), but no or negligible expression in blood cells. It is important to notice that the function of GLI2 can be dependent of the type of tissue in which it is expressed, but GLI2 is important for lung development [73] and altered expression might thus have an influence on the risk to develop respiratory allergies when *GLI2* methylation status is altered.

GLI2 is also suggested to be the primary GLI transcription factor transducing SHH-regulated lung growth. SHH is crucial in branching morphogenesis and formation of mature airways through branching events of bronchi and mesenchyme proliferation [68]. It is clear that in embryonic lung development, expression of SHH and its signaling molecules are highly regulated and SHH exerts its effect on different cellular components [68]. Rutter et al. [73] describe that *gli2*-null lungs show defective airway branching, left pulmonary isomerism, and severe lung hypoplasia. In combination with loss in cellular proliferation, this leads to a lung that is not able to support life at birth. Although there is a reduction in wet lung weight in *gli3*-null mouse, both *gli1*- and *gli3*-null mice still develop functional lungs. The analysis of different *gli*-null mice led to the suggestion that GLI2 primarily acts as a transcriptional activator of the lung [73]. It was also discussed that *Shh*-null mice have single lobed hypoplastic lungs, with decreased epithelium, mesenchyme, and malformations of the trachea. In addition, Rutter et al. showed that the levels of cyclins D1, D2, and E1 are linked to *GLI2* expression in the developing lung, suggesting that it may be involved in the regulation of cell cycle components. An overexpression of GLI2 resulted in an increase of these cyclins, leading to cellular proliferation and to an increase in wet lung weight [73]. Thus, when assuming a direct association between methylation and gene expression, one can speculate that suppression of *GLI2* is in association with a decreased expression of cyclins. As such, it can be postulated that *GLI2* hypermethylation is involved in the suppression of the cell cycle, with a possible negative effect on lung development and an increased chance of developing respiratory allergy.

## Conclusions

We found hypermethylation in the *GLI2* gene in saliva from school-age children with a respiratory allergy when compared to saliva from healthy control subjects. The interpretation of the biological effects of the observed *GLI2* hypermethylation is not straightforward because the gene is under different levels of control. Posttranscriptional modifications to GLI2A and GLI2R and feedback loops in the HH signaling pathway complicate the usually assumed one-to-one relation between hypermethylation and decreased gene/protein expression. We suggest that *GLI2* hypermethylation can trigger inflammatory pathways dependent on IL4 and TGF-β1 signaling. In addition, *GLI2* is one of the main downstream effectors of the SHH pathway, which plays a role in branching of the bronchi and immune system regulation.

Overall, this study warrants further investigation into the mechanism linking *GLI2* to respiratory allergic disease in order to highlight the importance of differential methylation of *GLI2* in respiratory allergy and to define its potential for early identification of individuals at risk for respiratory allergy development.

## Additional files

Additional file 1: **Figure S1.** Overview of chromatin state segmentation of the *GLI2* DMR. The blue-shaded area is the one covered by the *GLI2* DMR, including cg00637745, cg13872898, and cg17870997. This DMR region is enriched for H3K4Me1 and H3K27Ac histone marks, which are associated with enhancers or enhanced transcription, respectively. (PPTX 1138 kb)

Additional file 2: **Figure S2.** Gene expression in relevant tissues from GTEx RNA-Seq of the *GLI2* gene (hg19 chr2:121493199–121750229). Highest median expression was detected in the ovaries (8.16 RPKM), and total median expression was 71.60 RPKM. Significant expression was detected in lung (1.394 RPKM) and salivary gland (0.902 RPKM) tissues but was repressed in whole blood. (PPTX 440 kb)

Additional file 3: **Table S1.** Overview of DMRs in various respiratory allergy subtypes versus controls in the FLEHS1 birth cohort. **Table S2.** Overview of DMRs in house dust mite cases versus controls in the FLEHS2 birth cohort. (DOCX 17 kb)

## Abbreviations

ALOX12: Arachidonate 12-lipoxygenase
APOBEC1: Apolipoprotein B mRNA editing enzyme, catalytic polypeptide 1
CCR: Chemokine receptors
CD: Cluster of differentiation
DMRs: Differentially methylated gene regions
EFA: European Federation of Allergy and Airways Diseases Patients’ Associations
FLEHS: Flemish Environment and Health Studies
GATA3: GATA-binding protein 3
GLI: GLI family of zinc-finger DNA-binding proteins
GRAMD1B: GRAM domain containing 1B
H3K27Ac: Acetylated histone H3 at lysine 27
H3K4Me1: Monomethylated histone H3 at lysine 4
HH: Hedgehog
HTRA3: High-temperature requirement factor A3
IFN-γ: Interferon gamma
IgE: Immunoglobulin E
IL: Interleukin
IPA: Ingenuity Pathway Analysis
ISAAC: International Study of Asthma and Allergies in Childhood
MED24: Mediator complex subunit 24
NFκB: Nuclear factor kappa-light-chain-enhancer of activated B cells
NHLF: Normal Human Lung Fibroblasts
OPCML: Opioid-binding protein/cell adhesion molecule-like
PBMC: Peripheral blood mononuclear cells
RELA: Nuclear factor NF-kappa-B p65 subunit
RPKM: Reads per kb of exon model per million mapped reads
SHH: Sonic hedgehog
TGF-β: Transforming growth factor-β
Th: T-helper cell
