# Supplementary figures and images for: GLI2 promoter hypermethylation in saliva of children with a respiratory allergy
Source: Clin Epigenetics. 2018 Apr 11;10:50. doi: 10.1186/s13148-018-0484-1 (PMC5896137; doi:10.1186/s13148-018-0484-1)

## Slide 1
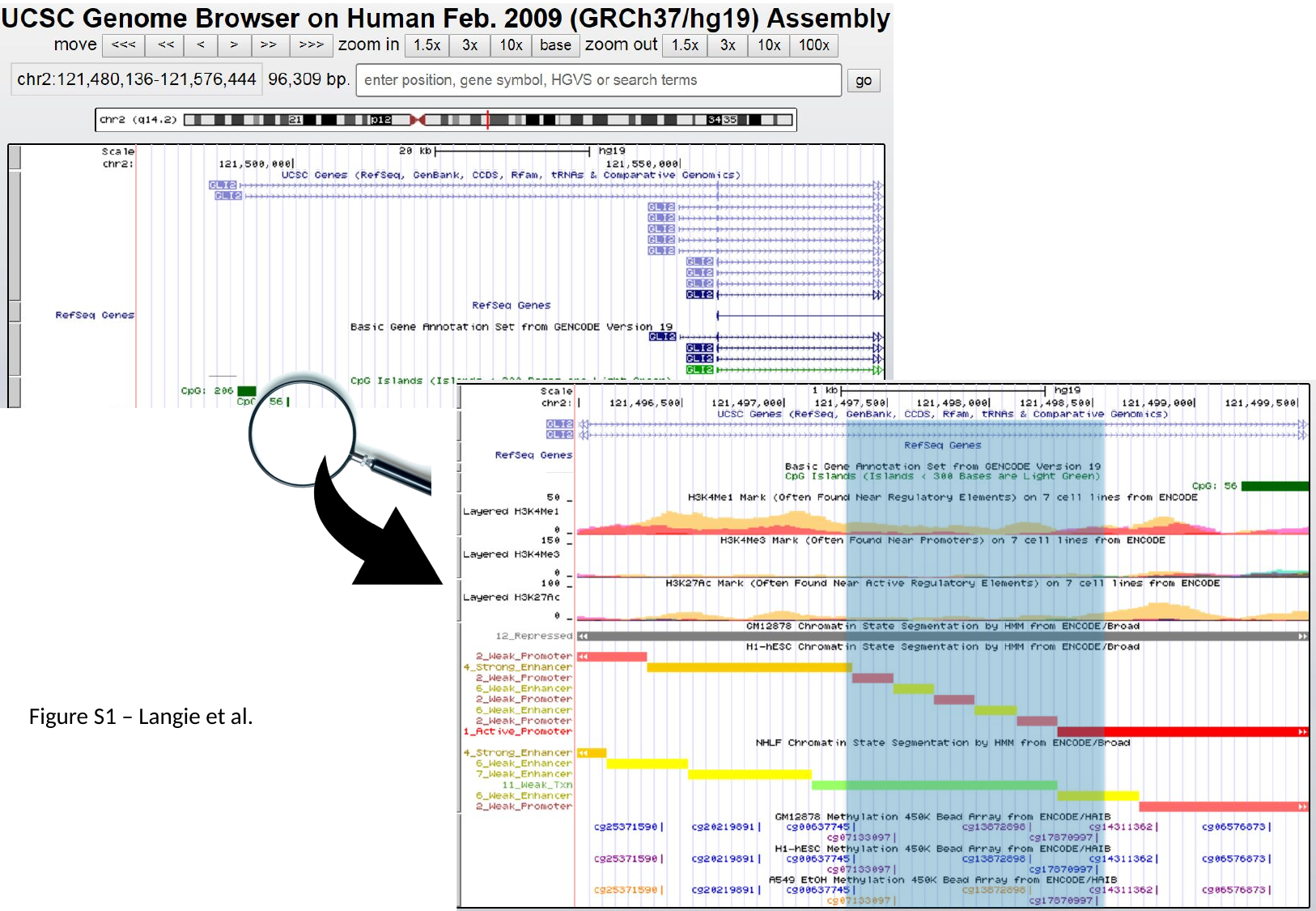

Figure S1 – Langie et al.

Supplement: Supplementary file 1 — Figure S1. Overview of chromatin state segmentation of the GLI2 DMR. The blue-shaded area is the one covered by the GLI2 DMR, including cg00637745, cg13872898, and cg17870997. This DMR region is enriched for H3K4Me1 and H3K27Ac histone marks, which are associated with enhancers or enhanced transcription, respectively. (PPTX 1138 kb) [file 13148_2018_484_MOESM1_ESM.pptx]
