# Supplementary material for: GLI2 promoter hypermethylation in saliva of children with a respiratory allergy
Source: Clin Epigenetics. 2018 Apr 11;10:50. doi: 10.1186/s13148-018-0484-1 (PMC5896137; doi:10.1186/s13148-018-0484-1)
Supplement: Supplementary file 2 — Figure S2. Gene expression in relevant tissues from GTEx RNA-Seq of the GLI2 gene (hg19 chr2:121493199–121750229). Highest median expression was detected in the ovaries (8.16 RPKM), and total median expression was 71.60 RPKM. Significant expression was detected in lung (1.394 RPKM) and salivary gland (0.902 RPKM) tissues but was repressed in whole blood. (PPTX 440 kb) [file 13148_2018_484_MOESM2_ESM.pptx]

## Slide 1
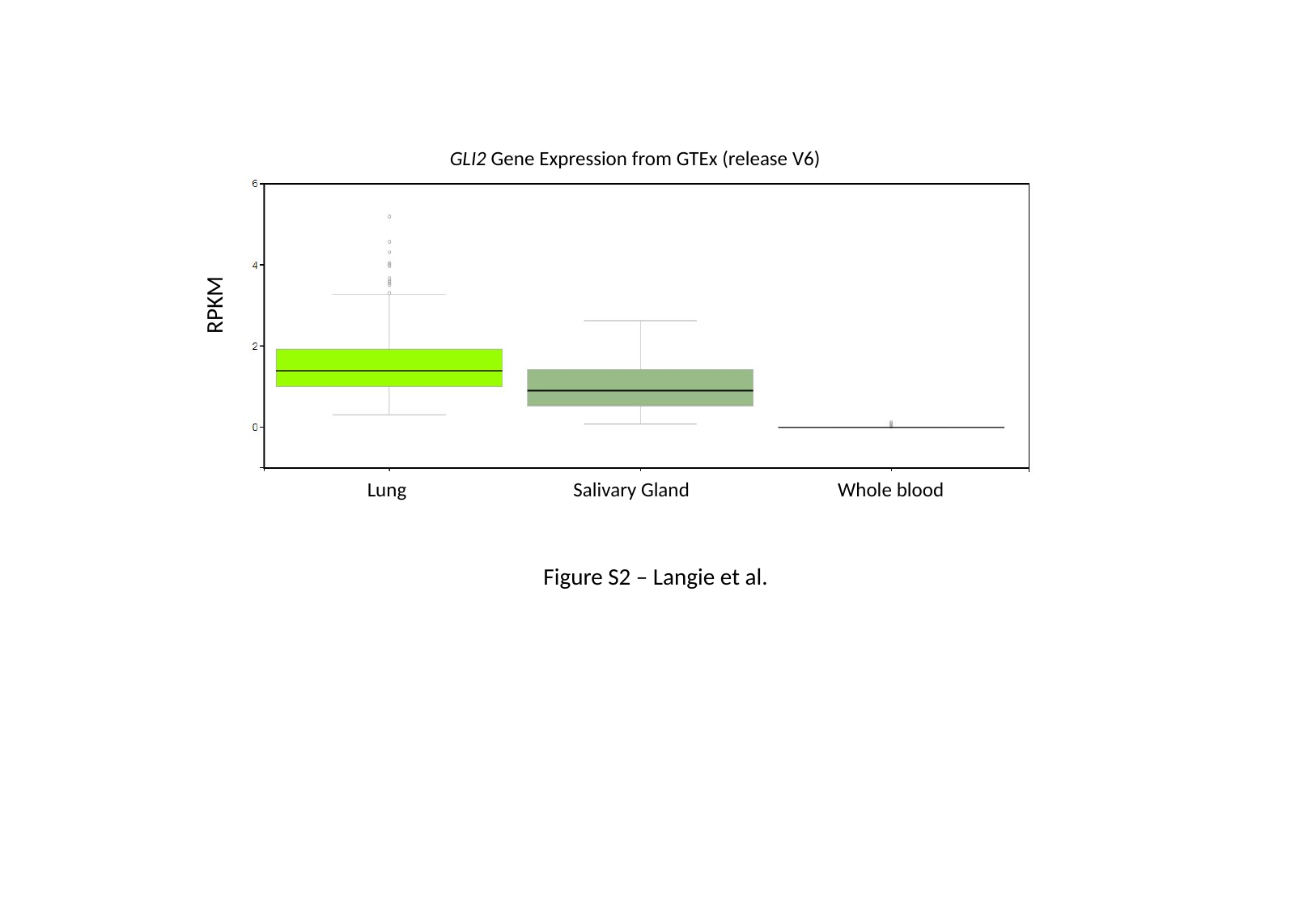

GLI2 Gene Expression from GTEx (release V6)
RPKM
 Lung Salivary Gland Whole blood
Figure S2 – Langie et al.
