# Supplementary material for: GLI2 promoter hypermethylation in saliva of children with a respiratory allergy
Source: Clin Epigenetics. 2018 Apr 11;10:50. doi: 10.1186/s13148-018-0484-1 (PMC5896137; doi:10.1186/s13148-018-0484-1)
Supplement: Supplementary file 3 — Table S1. Overview of DMRs in various respiratory allergy subtypes versus controls in the FLEHS1 birth cohort. Table S2. Overview of DMRs in house dust mite cases versus controls in the FLEHS2 birth cohort. (DOCX 17 kb) [file 13148_2018_484_MOESM3_ESM.docx]

Table S1: Overview of DMRs in various respiratory allergy subtypes versus controls in the FLEHS1 birth cohort.

| Correction method \ Subtype | Asthma (N=8) | | | Hayfever (N=16) | | | Rhinitis (N=23) | | |
| --- | --- | --- | --- | --- | --- | --- | --- | --- | --- |
|  | Gene | Δβ (%) | p-value | Gene | Δβ (%) | p-value | Gene | Δβ (%) | p-value |
| No cell correction |  |  |  | *ALOX12* | -5.49 | <0.001 |  |  |  |
|  |  |  |  | *APOBEC1* | -3.46 | 0.003 | *APOBEC1* | -2.69 | <0.001 |
|  | *GLI2* | -3.47 | <0.001 |  |  |  | *GLI2* | 1.04 | <0.001 |
|  | *OPCML* | -4.37 | 0.001 | *OPCML* | 1.35 | 0.017 |  |  |  |
| Reference based correction according to Houseman et al. | *ALOX12* | -3.01 | 0.002 | *ALOX12* | -5.49 | <0.001 |  |  |  |
|  |  |  |  |  |  |  | *APOBEC1* | -2.69 | 0.006 |
|  |  |  |  |  |  |  | *GLI2* | 1.04 | <0.001 |
|  |  |  |  |  |  |  | *GRAMD1B* | 1.04 | <0.001 |
|  |  |  |  |  |  |  | *MED24* | -1.12 | <0.001 |
|  |  |  |  |  |  |  | *OPCML* | 0.87 | 0.015 |
| Corrected for granulocyte and buccal cell proportions | *ALOX12* | -3.01 | <0.001 | *ALOX12* | -5.49 | <0.001 | *ALOX12* | -4.21 | 0.002 |
|  |  |  |  | *APOBEC1* | -3.46 | <0.001 | *APOBEC1* | -2.69 | <0.001 |
|  |  |  |  | *GLI2* | 2.40 | <0.001 | *GLI2* | 1.04 | <0.001 |
|  |  |  |  | *OPCML* | 1.35 | 0.004 | *GRAMD1B* | 1.04 | 0.015 |
|  |  |  |  |  |  |  | *MED24* | -1.12 | 0.013 |
|  |  |  |  |  |  |  | *OPCML* | 0.87 | 0.003 |

Table S2: Overview of DMRs in house dust mite cases versus controls in the FLEHS2 birth cohort.

| FLEHS2 - iPLEX | House dust mite (N= 5 cases vs. 20 controls) | | |
| --- | --- | --- | --- |
|  | Gene | Δβ | p-value |
| No cell correction | *GLI2* | 10.82 | 0.058 |
|  | *GRAMD1B* | -2.79 | 0.050 |
|  | *HTRA3* | 7.30 | 0.011 |
|  | *OPCML* | -11.31 | 0.042 |
